# Reorienting Primary Health Care Services for Non-Communicable Diseases: A Comparative Preparedness Assessment of Two Healthcare Networks in Malawi and Zambia

**DOI:** 10.3390/ijerph18095044

**Published:** 2021-05-10

**Authors:** Veronica Shiroya, Naonga Shawa, Beatrice Matanje, John Haloka, Elvis Safary, Chikondi Nkhweliwa, Olaf Mueller, Sam Phiri, Florian Neuhann, Andreas Deckert

**Affiliations:** 1 Heidelberg Institute of Global Health, Im Neuenheimer Feld 130.3, 69120 Heidelberg, Germany; elvis.safary@uni-heidelberg.de (E.S.); Olaf.Mueller@urz.uni-heidelberg.de (O.M.); florian.neuhann@uni-heidelberg.de (F.N.); a.deckert@uni-heidelberg.de (A.D.); 2 Health Promotion Alliance of Kenya, Trans Nzoia County, Kitale 30200, Kenya; 3 CHRESO Ministries, Lusaka 10101, Zambia; naonga.shawa@gmail.com (N.S.); jpoto75@yahoo.com (J.H.); 4 The Lighthouse Trust, Kamuzu Central Hospital, Lilongwe 207233, Malawi; bmatanje@lighthouse.org.mw (B.M.); cnkhweliwa@gmail.com (C.N.); samphiri@lighthouse.org.mw (S.P.); 5 School of Medicine and Clinical Sciences, Levy Mwanawasa Medical University, Lusaka 10101, Zambia

**Keywords:** health systems, implementation research, mixed methods study, low-income countries, chronic disease control, human resources for health, primary care, health policy, health service delivery, Sub-Saharan Africa

## Abstract

Despite positive NCD policies in recent years, majority of Sub-Saharan African (SSA) health systems are inadequately prepared to deliver comprehensive first-line care for NCDs. Primary health care (PHC) settings in countries like Malawi and Zambia could be a doorway to effectively manage NCDs by moving away from delivering only episodic care to providing an integrated approach over time. As part of a collaborative health system strengthening project, we assessed and compared the preparedness and operational capacity of two target networks of public PHC settings in Lilongwe (Malawi) and Lusaka (Zambia) to integrate NCD services within routine service delivery. Data was collected and analyzed using validated health facility survey tools. These baseline assessments conducted between August 2018 and March 2019, also included interviews with 20 on-site health personnel and focal persons, who described existing barriers in delivering NCD services. In both countries, policy directives to decentralize disease-specific NCD services to the primary care level were initiated to meet increased demand but lacked operational guidance. In general, the assessed PHC sites were inadequately prepared to integrate NCDs into various service delivery domains, thus requiring further support. In spite of existing multi-faceted limitations, there was motivation among healthcare staff to provide NCD services.

## 1. Introduction

In the last four decades, the global discourse around primary health care (PHC) highlighted its critical role in most national health systems. For non-communicable diseases (NCD), PHC could be a doorway to effectively manage chronic diseases by moving away from delivering only episodic care to providing an integrated approach that includes prevention, diagnosis, treatment, and palliative care for all conditions and over time [1,2]. Consistent with the trend in many African countries, Malawi and Zambia are facing similar disease profiles; the threat of a rapidly growing burden of NCDs and injuries, and efforts to address them are overshadowed by existentially high burden of infectious diseases like HIV/AIDS, malaria etc. and reproductive, maternal and child health (RMNCH) conditions. According to the Malawian Ministry of Health (MoH) in 2017, NCDs in the country accounted for at least 12% of total disability adjusted life years (DALYs) and at 16%, are the second leading cause of death in adults after HIV/AIDS [3,4]. NCDs in both countries disproportionately affect young people, with up to 60.5% of NCD DALYs constituting people under the age of 40, as compared to 18.5% in high- income countries [5]. Recognizant of this triple threat (infectious diseases, NCDs and poverty), both countries form part of a global NCDs and Injuries Poverty Commission to further understand the unique linkage between NCDs and high poverty burden and its implications for effective response in the affected demographics [3,4,6].

The health care systems in Malawi and Zambia are similarly structured into three-tiered service delivery levels, offering primary, secondary and tertiary healthcare. The primary level comprises of community initiatives, health posts, dispensaries, maternity facilities, health centres and community/rural hospitals. The secondary level entails district hospitals (Malawi) or provincial and general hospitals (Zambia) while the tertiary level services are provided by the central hospitals and national university teaching hospitals. The three levels in both healthcare systems are linked by a referral system with the central and university teaching hospitals being at the top [7,8].

The disease burden in Malawi and Zambia and profile of key determinants of health, along with respective milestones during the millenium development goals (MDG) era, are to a greater extent anchored in the performance of their PHC systems where majority of the population access the health system [9,10,11]. The tiered structure of both health systems means that their PHC networks at the lowest level, are directly linked to community and household structures. This highlights their important role of serving last mile populations, the majority of whom live in rural areas (84% and 57% in Malawi and Zambia respectively) and for urban areas, the low-income segments of the population, e.g., slums and informal settlements [12,13]. This also justifies the inclusion of NCDs among priority public healthcare services within the respective essential packages for health outlined in health sector-related policies in both countries [14,15]. However, existing evidence, albeit limited, highlights discrepancies between the presence of such policies and the realities of their implementation. In 2017, a report by the World Health Organization (WHO) on worldwide surveys on country capacity for NCD prevention and control showed that 78% of Sub-Saharan Africa (SSA) countries reported having integrated national NCD plans. However, only 32% of these plans were operational, covering the four main conditions (cardiovascular diseases, diabetes, chronic obstructive pulmonary diseases (COPD)/asthma and cancer types) and their risk factors [16]. Since 2010, only 28% of SSA countries have conducted recent national or sub-national surveys of health facilities to assess service availability and readiness for NCDs. Current evidence also highlights that majority (up to 94%) lack the necessary equipment generally available to take six essential primary care measurements: height, weight, blood glucose, blood pressure, total cholesterol, and urine albumin [1,17].

Following the 2011 United Nations declaration on NCDs, Malawi and Zambia developed and launched national action frameworks for NCDs in 2013 [18,19]. These were aligned to a global NCD action plan with 9 voluntary targets [20]. Malawi and Zambia also adopted the WHO package of essential NCDs (WHO PEN) interventions for low-resource primary care settings to guide implementation alongside respective national essential drug lists [21,22,23]. Studies that have analyzed NCD policies in Malawi and Zambia show that these existing strategies meant to provide a road map for addressing NCDs locally were initiated by the governments through respective MoH department-led efforts, but lacked effective multisectoral involvement [24,25,26]. As a result, the domestication of NCD policies remains a challenge as they are heavily reliant on international frameworks and experience inadequate political commitment, resources and technical capacity [25]. A recent shift to local evidence building for NCDs to inform policy reforms requires an understanding of operational processes at all levels of care, including primary health care settings. Among policy priority areas for improving NCD services and technical capacity at PHC levels in both countries is the recruitment, training and retention of health workers. Zambia’s National Health Strategy 2017–2021, estimates a human resource gap of 32% nationally in 2016 [15]. This is almost similar to Malawi’s 33% gap [14]. The situation is more dire at PHC level where on average, both countries operate at less than 50% human resource capacity due to shortage of clinical officers, nurses and midwives. Moreso, community health workers responsible for patient follow-up roles and health promotion also experience high attrition rates due to unfavorable working conditions [12,27].

The study described in this paper feeds into the larger objectives of a health systems capacity strengthening initiative for implementation of evidence-based integration models for NCDs in Zambia and Malawi. The Zambia–Malawi Collaboration (ZaMaC) on NCDs aims to prove the patients’ direct benefit of accredited continuous medical education (CME) of healthcare staff and comprehensive capacity strengthening measures to promote national universal health coverage efforts [28]. The collaborating institutions under ZaMaC Project are recognized by the governments of Zambia and Malawi. They also leverage their linkage to other existing networks locally and abroad in research, education and other sectors to advance collaborative approaches to addressing NCDs. As part of this initiative, we performed baseline assessments prior to the project interventions, targeting selected primary care settings with respect to the state of NCD service integration within routine service delivery in the respective countries. The study addressed in this paper include the results of the baseline assessments, which explored existing gaps in reorienting NCD care at PHC level from the perspective of health service provision at implementation (facility) level.

## 2. Materials and Methods

### 2.1. Study Setting

**Figure 1:**
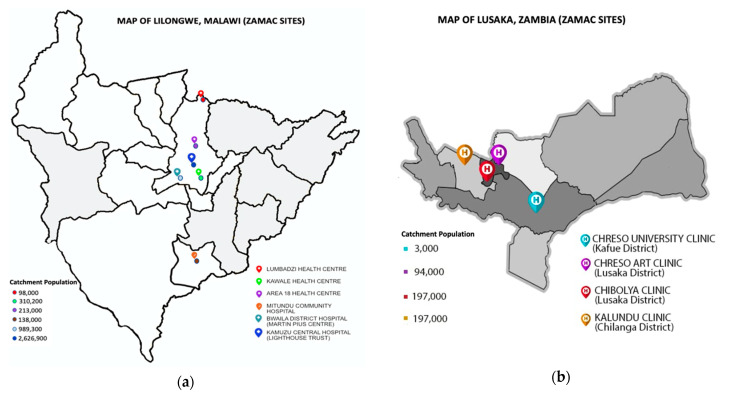
Locations (**a**,**b**) of target primary healthcare sites and their catchment populations [31].

Under the Zambia–Malawi Collaboration on NCDs project, two collaborating institutions in the respective countries have a network of 8 public PHC facilities each in Lusaka and Lilongwe that they support in first line healthcare provision, referred to as “prototype sites” (Malawi) or ‘‘satellite sites” (Zambia). The Lighthouse Trust in Malawi is a registered public trust, originally founded to support sector-wide efforts towards a coordinated national response to HIV/AIDS in Malawi. It is also a WHO recognized Centre of Excellence for HIV/AIDS research and quality care provision in the country [29]. Similarly, in Zambia, CHRESO Ministries is part of the country’s network of faith-based organizations that support the national response efforts for HIV/AIDS and other health and education sector initiatives [30]. Our sample frame for the study were the catchment areas served by the aforementioned PHC networks in Lilongwe, Malawi and Lusaka, Zambia. These are distributed in urban, peri-urban and rural sections of the target districts. As at 1st January 2018, the assessed primary care sites served a combined catchment population of 1,112,200 of which Lilongwe sites (Area 18, Lumbadzi, Mitundu and Kawale) constituted 621,200 (55.9%) and Lusaka sites (Chibolya, CHRESO ART (antiretroviral therapy) centre, CHRESO university and Kalundu) constituted 491,000 (44.1%) [Figure 1]. All sites proximally target health services to disadvantaged populations, i.e., urban sites for low-income informal settlements and rural sites for hard-to-reach rural populations. The assessed facilities in Malawi were government-owned public facilities while Zambian facilities were faith-based supported public facilities.

### 2.2. Study Objectives (Research Questions)

Our main study objective was to assess the operational capacity of selected primary care settings in Lilongwe district and Lusaka Province towards integrating routine NCD services. We sought to answer the following:

- What is the current situation of NCD service delivery within target facilities in the respective regions?
- What is the status of human resource for NCD service delivery?
- What are current barriers in implementing NCD service delivery models in the respective primary healthcare settings?

### 2.3. Study Design

**Figure 2:**
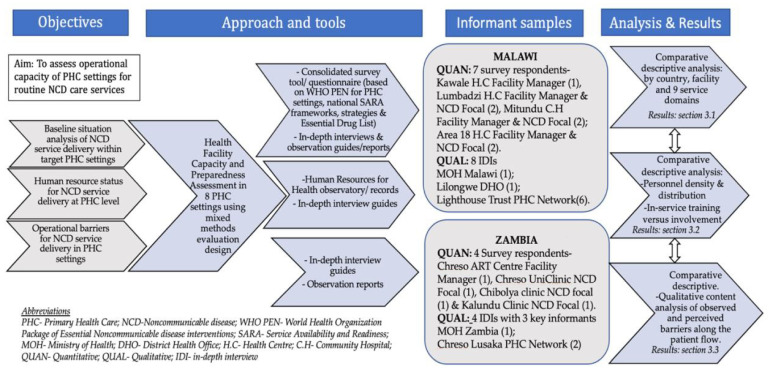
Study theoretical and methodological framework.

To address the study objectives, a cross-sectional concurrent mixed methods design as exemplified by Schoonenboom and Johnson [32] and Patton [33] was employed and translated in a framework as depicted in Figure 2. This is where the quantitative survey component was consolidated with qualitative components including interviews, observation notes and review of reconnaissance visit reports to obtain a holistic picture of the facility context. We also utilized approaches proposed by Beran and Higuchi [34] and Patton [33] to guide the operationalizing of our data collection tools and assessment process [33,34]. Both approaches offer guidance for optimizing data collection for NCDs (Beran and Haguchi) in a utilization-focused environment such as a health facility (Patton). The reconnaissance investigations happened between December 2017 to March 2018 to map out facilities served by the network of both institutions.

### 2.4. Study Sample and Recruitment of Participants

For each country network, we purposively selected four out of eight facilities comprised in each respective network of PHC facilities in Lilongwe and Lusaka described in Section 2.1. The criteria for facility selection were (i) the facility was designated for primary care services; (ii) the PHC facility was a prototype or satellite site as described in Section 2.1; (iii) the PHC facility was accessible to the public and; (iv) the facility management consented to the assessment. For each site, respective facility managers or NCD/departmental focal persons were also approached for consultative interviews accompanying the interviewer-administered surveys. These were staff, at least 18 years old, stationed or seconded to the health settings and with decision-making responsibilities on behalf of the target sites. The approach used was snowball sampling and only key informants who consented to the interviews were included in the study sample (Figure 2). A minimum of one survey respondent per facility site was considered sufficient to achieve the study objectives.

### 2.5. Data Collection

Both countries have contextualized service provision assessment (SPA) tools and the WHO service availability and readiness assessment (SARA) tool [7,35,36]. However, as these tools are often developed for wider scale national surveys, we adopted sections of the WHO SARA, WHO PEN implementation tools, Zambia and Malawi SPA questionnaire and reorganized them for a more-in-depth assessment of individual health facility context covering the following sections:

(i) the general description of services and human resources;
(ii) infrastructure and basic equipment/technologies;
(iii) essential medicines;
(iv) utilization of services (and referral of patients);
(v) community outreach and linkage to facility.

To further validate our hybrid questionnaire, we piloted all study tools and consulted experts to gather local inputs on their relevance before adopting them for the formative assessment. For each section of the questionnaire, we probed and confirmed for availability of the items and measures listed on the checklists. A list of all items and measures can be found on Table A1. For processes, participatory observation at the point of care by the investigator was done to outline and illustrate the patient flow process, included hereafter in results Section 3.3.2. The quantitative and qualitative aspects of the questionnaire were obtained from informants as outlined in Figure 2. Their views on their individual capacity and working conditions with regards to NCD care were noted. They also helped to clarify observations and give context to the preliminary results of the assessment, hence validating the survey further. The primary data collection at the sites was conducted over a period of three to four weeks with two to three visits per site per country. The baseline assessments in both countries happened between August 2018 and March 2019.

### 2.6. Data Analysis and Dissemination

For quantitative data, we used a scoring matrix with components such as rates of service availability, human resource, drug availability, etc. The matrix yielded 78 tracer items in 9 service domains (Table A1). Our operational definitions were as follows:

- Integration: To which extent disease-specific services for NCDs (such as screening, primary management, referral pathways) are embedded within routine general outpatient services including but not limited to ART services.
- Operational capacity: The availability of tracer items confirmed per assessment domain as pre-requisites for NCD service delivery.
- Preparedness: To which extent an assessed facility meets the calculated sum of required value scores for service preparedness and compares with the existing national or international requirement for a service domain, e.g., for human resource requirements.

**Figure 3:**
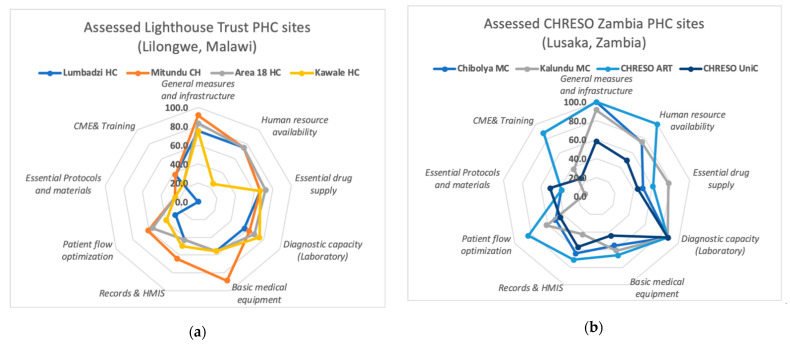
Spider graphs showing capacity score (by basic service domain requirements) for NCD service integration by assessed PHC sites in Lilongwe (**a**) and Lusaka (**b**).

We calculated our working benchmark/required values score for each service domain by comparing tracer items from the most recent international, national or district capacity survey tools [7,16,35]. Each domain score was calculated as the mean score of items as a percentage [36]. We then summed up the individual service domain scores against a total capacity score of 78 (100%) to obtain our working benchmark cut-off for NCD service preparedness. The capacity score per country network was calculated as the mean of the sum of weighted service-specific preparedness scores across the respective PHC sites. The calculations were done using MS Excel. We summarized the results of this analytic approach in a matrix Table A1 and for individual facilities, Figure 3 in results section. We triangulated quantitative results with content analysis of transcribed interviews and observation notes to guide the explanation of our assessment results. Exemplary opinions were quoted for emphasis. To guide our explanatory flow of results between the two countries, we used a narrative approach in three results Section 3.1, Section 3.2 and Section 3.3 based on identified themes under each objective (Figure 2). We also presented findings in distribution diagrams and tables to highlight some of the main similarities and differences noted between the assessed sites within and between the two countries.

### 2.7. Ethical Considerations

The participation of respondents and facilities during this study was voluntary. Within facilities, prior familiarization visits were conducted to explain study objectives and on assessment day, verbal and written informed consent was obtained from facility managers and health workers. To ensure confidentiality of information, all collected personal data were anonymized. Ethical clearance was sought via the Lighthouse Trust and CHRESO Ministries. In addition, the first author obtained separate approval from the ethical committees of Heidelberg University (S-345/2018) and the Malawi National Health Service Research Commission (NHSRC-18/07/2073). The results of the formative assessment were subsequently disseminated to local stakeholders.

## 3. Results

### 3.1. Current Situation of NCD Service Delivery in Lilongwe and Lusaka

#### 3.1.1. Context and Availability of Integrated Priority NCD Services

Health services in all assessed PHC facilities were available to the public for free through government or donor subsidies. All facilities in both country network sites ran routine antiretroviral therapy (ART)-related services. Integrated service delivery for uncomplicated NCDs in the respective primary care sites in Malawi was still in early stages (less than 12 months operational). According to the Lilongwe District Health Office (DHO), the four primary care sites we assessed in our study sample of Lighthouse Trust’s PHC network also constituted four out of six (67%) of public PHC facilities currently offering NCD services in the entire district (as at 31st December 2018). The corresponding service-specific coverage information for our Zambia sample was not available in the reviewed public records. According to respondents, NCD services at PHC level in Lilongwe were officially introduced in November 2016 in response to the overburdened NCD clinics at tertiary level in the main central hospital. In the assessed sites in Lusaka, availability of NCD services at primary care level had stemmed initially from routine monitoring of clients attending ART clinics in the respective sites. These services for uncomplicated NCDs had been operational for up to 24 months prior to the assessment day. However, service delivery was reported as mainly ad hoc with no systems or tools in place to monitor or measure progress. According to respondents, previously, suspected NCD clients were referred to the nearest secondary facility for further review, treatment and follow-up but increased demand led to reorientation of such services to include non-ART clients. Among four priority NCD services (excluding mental health and injuries) identified in both country networks (cardiovascular diseases, diabetes, asthma and cervical cancer screening) routine services for hypertension and asthma were available in all assessed facilities (Table 1), [15] (pp. 42–44), [14] (pp. 24–25).

#### 3.1.2. Comparative Assessment of Facility Capacity as per NCD Service Domains

As outlined in the matrix Table A1, our analysis yielded 53 (67.9%) as our working value/benchmark score for assessing current service preparedness in the respective sites in both countries. Baseline results show that on average, the assessed PHC sites in both country networks were generally less prepared to deliver standard NCD services as they did not attain this benchmark score. While none of the facilities attained the target score of 78 (100%) (Table 2, row 7), CHRESO Lusaka (Zambia) PHC sites attained a marginally higher average capacity score of 49.4 (63.1%) than the Lighthouse Lilongwe (Malawi) PHC sites with 46 (59%). Table 2 further exemplifies that Zambian facilities showed better preparedness in terms of availability of assessed tracer items and measures in most NCD service domains or sub-domains with the exception of tracer items in basic medical equipment and essential NCD drugs, where PHC sites in Malawi were marginally better prepared.

#### 3.1.3. The Operational Situation of NCD Services in Individual Health Facilities

The spider graphs (Figure 3) show that within sub-domains, individual facilities varied in attainment of basic prerequisites for NCD service provision. In terms of general infrastructure, all but one facility met the required score (66.7%) for general measures and infrastructure as an assessed domain for NCD service provision (Figure 2, Table 2). At the operational level, checklists and respondents cited challenges in equipment maintenance and usage, such as lack of batteries for blood pressure measuring devices, unavailable or wrong size BP cuffs, missing parts (nebulizers, spacers for inhalers, etc.). Frequent shortages in lab reagents, urine and glucose sticks were also reported. On the positive, health workers expressed support for decentralized services for NCDs but bemoaned the lack of operational guidance and this finding is also supported by the spider graph which shows generally less preparedness not only for human resource availability but also in terms of their training, lack of clinical protocols and for Malawi facilities, and the poor management of health records.

**Figure A1:**
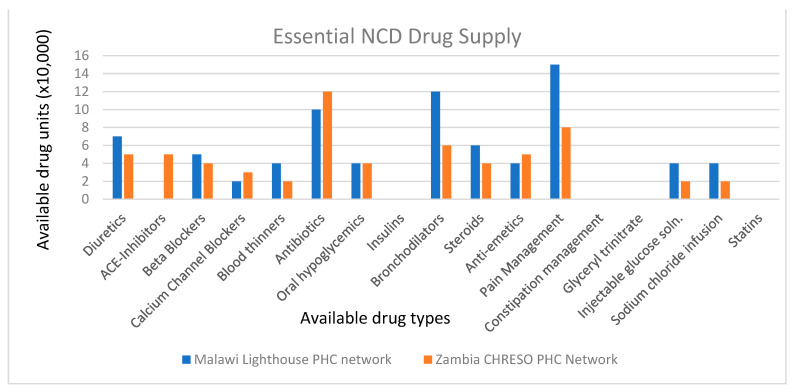
Availability of essential NCD medicines (by 18 drug types) in the assessed PHC sites.

Another recurring theme as per respondents’ perceptions in Table 3, was the persistent challenge of drug supply with frequent stock outs reported and other operational challenges in supply chain optimization and drug dispensation. None of the facilities including medical officer-led ones such as Mitundu (Malawi) and CHRESO ART (Lusaka) stocked insulin at the time of the assessment, citing a lack of proper storage facilities (Figure A1). As a result, despite government subsidy of drugs in all facilities, patients are often asked to purchase drugs via private avenues. In CHRESO Zambia, the designated supply of drugs from public sources could not meet the existing demand, hence reliance on mostly donor and philanthropic sources (Table 3, row3). In Malawi, drug supply was entirely via government (public) procurement.

### 3.2. Human Resources for NCD Service Delivery

The combined skilled health worker density for the assessed facilities in Malawi and Zambia are 1.7 and 0.4 per 10,000 catchment population respectively which was below both the national and global benchmarks (4.7 and 25.2 per 10,000 population for Malawi and Zambia respectively; 44.5 per 10,000 globally) [17,37]. This finding was consistent with some respondents’ accounts of low motivation and poor attitude towards NCD services among health personnel due to reasons such as “knowledge gaps and lack of training” and “priority to maternity and HIV/AIDS clients” and “perception of NCDs as extra work burden” (Table 3, Rows 4–9).

**Figure 4:**
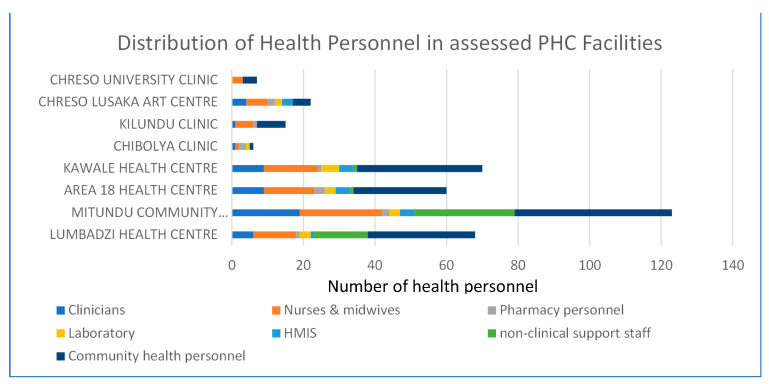
Distribution of health personnel in assessed PHC Facilities by cadre.

According to facility managers, the assessed sites met the basic threshold of two designated personnel (one of whom is a clinician) for NCD service provision. With the exception of CHRESO university site, this result is also exemplified in Figure 4. The distribution chart also confirms that Lilongwe sites had higher density of clinical and non-clinical health personnel than Lusaka sites. As at the assessment period, 6 facilities (3 Malawi: Kawale, Lumbadzi, Area 18 and 3 Zambia: Kalundu, Chibolya, Chreso UniClinic) were clinical officer-led while 2 facilities (Mitundu, Malawi and Chreso ART, Zambia) were medical officer-led. In both country contexts, clinical cadres are responsible for patient review, management and drug prescription while nurses and clinic aides dispense prescriptions, do patient follow-up and health education. Nurses are also responsible for triaging at outpatient waiting areas. With the exception of CHRESO ART (Zambia), health workers in all clinical sites had not received a refresher NCD training (for hypertension, diabetes or asthma) in the previous 24 months.

In Zambia, the set-up of services was aligned to daily ART services or general outpatient services. This enabled provisions for NCD services to be offered concurrently at the same point of care among available designated health workers. This was in contrast to Malawi where NCD services in all assessed sites but one were solitary clinics tied to one or two clinicians, whose absence severely interrupted routine service delivery (Table 3, Rows 5,6). Additionally, it was noted that the Zambian sites, nurses and midwives were mostly involved in NCD services, while in Malawi, nurses including those reported to have been previously trained or oriented were almost entirely uninvolved in NCD clinics. The reason cited for this was a greater need to prioritize their availability in RMNCH services. In two PHC facilities (CHRESO ART Centre, Zambia and Lumbadzi Health Centre, Malawi) that operated NCD clinics alongside ART services, we noted the presence of non-clinical staff supporting roles such as registration, vital check and patient documentation.

We also observed that other important cadres such as data clerks and pharmacy personnel were generally not involved, trained or oriented for NCD service delivery support. Consequently, such corresponding NCD-related responsibilities were delegated to one staff, often clinicians with other responsibilities. This heavy workload burden on clinicians resulted in lengthy waiting times for NCD clients. This discrepancy also reflected in the management and coordination of NCD-related drug and equipment supply and facility-level data management practices, which were sub-optimal.

### 3.3. NCD Service Delivery and Barriers along the Patient Flow Pathway

#### 3.3.1. Service Utilization and Data Management

**Figure A2:**
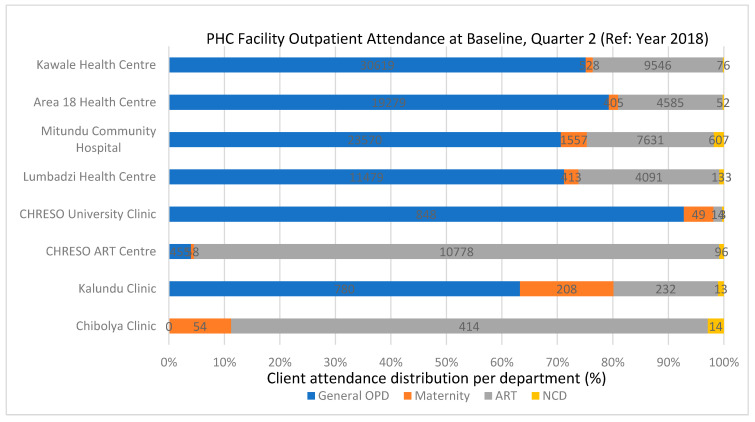
Reported average patient attendance per facility during second quarter 2018 (note: general outpatient department (OPD) figures exclude NCD outpatient attendance).

Facility records at baseline show attendance for NCD services was evidently low compared to other departments but as previously noted, reporting practices for NCDs were also inconsistent (Figure A2). In most instances (all 4 assessed Malawi sites and 1 Zambia site), disaggregation of reports into indicators such as sex, age and NCD type was not done or captured into registers. All assessed facilities in both countries also reported at baseline, that district supervision visits for NCDs had not been done since inception of the services. NCDs were also yet to be integrated into district reporting requirements. In Zambia, NCDs were only reported in the context of HIV/AIDS services. According to facility managers, the existing electronic system in use (SmartCare) for national and sub-national lacked provision for NCD reporting. This is due to restrictions in the system development that limit the end-user’s ability to analyze or generate customized NCD reports for planning or decision-making purposes. In Malawi, a recently launched electronic system of NCD reporting was yet to be operationalized in 2 of the 4 assessed facilities and remained unutilized in the remaining 2 facilities. All assessed facilities in both countries therefore relied entirely on manual record keeping for NCDs.

#### 3.3.2. Patient Flow

**Figure 5:**
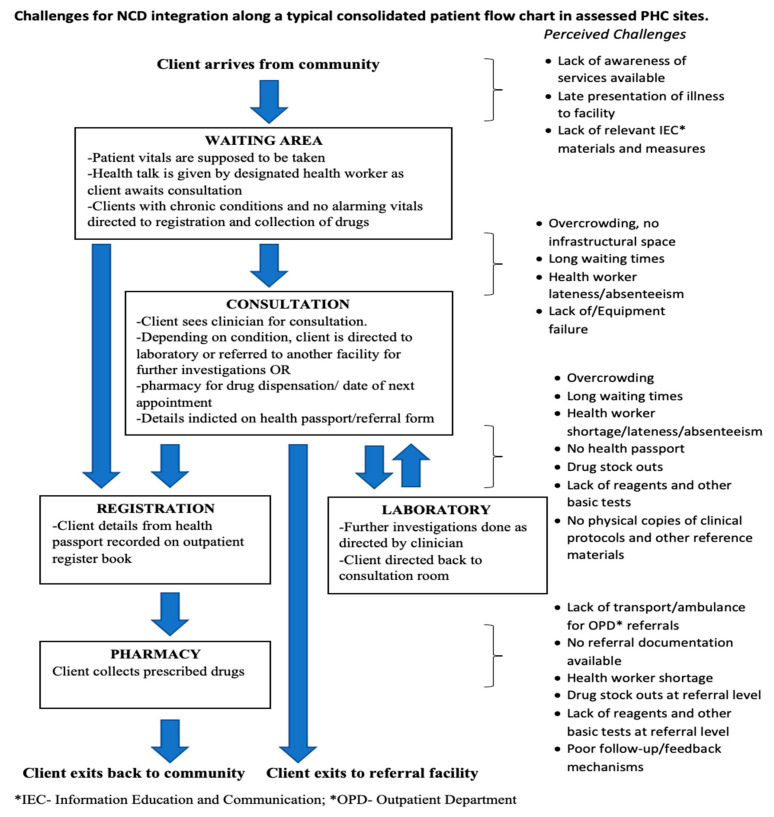
Description of operational challenges for NCD integration along a typical consolidated patient flow in assessed PHC sites.

All primary care facilities had no guiding signage to reflect availability of NCD services. Clients were either referred verbally by staff or fellow patients. Figure 5 outlines a typical consolidated patient flow chart in assessed sites outlining challenges along service delivery points. Alongside challenges faced in the facilities, NCD clients also lacked support groups/mechanisms to strengthen health promotion, monitoring and follow-up outside the facilities. Nevertheless, respondents had positive views of integrated NCD clinics for the potential benefit of more effective management and care of clients. Besides the need to step up community awareness of available NCD services, some respondents perceived “one-stop shop” as a preferred approach for better optimization along the patient flow (Table 3, Row 8). Table 3 highlights further verbatim accounts of respondents’ experiences in integrating NCD services.

## 4. Discussion

### 4.1. Summary of Findings

This paper highlights the results of baseline assessments targeting selected public (government-ran and faith-based supported) primary care settings serving a healthcare network of two collaborative HIV/AIDS-led institutions in Lilongwe, Malawi and Lusaka, Zambia. Our baseline situational analysis of the assessed PHC contexts in Malawi and Zambia revealed that integration of NCD services at PHC level were in early stages but the services in Zambian sites had been operational longer (generally 24 months) than Malawi (generally 12 months). The services in Malawi stemmed from the need to decongest overburdened tertiary facilities while in Zambia, NCD services were mostly anchored in ART services whereby increased demand made it necessary to integrate non-ART clients. As a result, we noted that Zambian sites (63.1% score) showed marginally better preparedness for NCD service integration compared to Malawian sites (59% score). However, on average, both networks were generally below par (67.9% benchmark score). Based on our findings, both country contexts experience mostly similar operational barriers. The most notable was the lack of operational guidance to deliver NCD services to the public which translated to low prioritization of the services. The quality and uptake of NCD related data was also generally poor. Furthermore, our assessments also highlighted that government support in reorienting the infectious-disease oriented PHC systems in both country contexts towards NCD services was generally insufficient. These results were quantified further by disparities in the availability of basic medical equipment and diagnostic capacity, erratic essential NCD drug supply, weak patient flow and referral mechanisms. We further highlighted discrepancies in the training versus involvement of existing health worker cadres for NCD-related PHC services in both contexts. None of the existing health workers in all but one site had received refresher training the previous 24 months. Worth noting from our qualitative results, was that in spite of prevailing challenges, PHC-level health workers expressed motivation to deliver NCD services.

### 4.2. Early Lessons and Operational Implications for Malawi and Zambia and Similar PHC Settings

#### 4.2.1. Addressing Existing Service Delivery Gaps for NCD Integration at PHC Level

For our analytical approach, the 67.9% benchmark score used was close to 70% used in other studies [35]. The operational gaps highlighted in our study have previously been identified in other low-resource contexts that have utilized the WHO SPA, WHO SARA and WHO PEN methodologies [7,16,35,38,39,40]. We identified among other capacity gaps, the recurring challenge of unavailability and erratic supply of essential NCD drugs and basic medical equipment in both assessed health networks in Malawi and Zambia. A separate study in Zambia [41] outlined reasons for elevated stockouts of essential medicines in health facilities to include; unsystematic and unpredictable secondary distribution channels between district medical stores and health centres, ineffective communication channels, lack of demand data which often do not take actual consumption patterns into account, transport challenges and lack of human resources for logistics and monitoring purposes. For our assessed sites in Malawi, these findings correspond with respondents’ accounts of inefficient and ineffective NCD-related drug and equipment supply practices that fail to meet contextual operational needs of PHC settings. It is worth acknowledging that the problem of erratic drug supply is often underpinned by complexities beyond the facility level and exercabated further by a complex political economy that is characteristic of hierarchical medical supply chain systems in such contexts. Therefore, we recommend that the health networks in Zambia and Malawi referenced in our study, leverage further the existing multi-sector collaborative platforms to integrate and address thematic issues in NCD service delivery within their contexts. At the operational level, the existing PHC networks and other settings with similar contextual challenges could adopt measures like assigning pre-packaged NCD medications, which based on previous studies [41,42], could facilitate speedy distribution to clients to avoid lengthy waiting times. As they are less cost-intensive, challenges in maldistribution of basic equipment like stationery, IEC materials, standardized reporting, inter-facility linkages and referral mechanisms could be addressed through locally driven measures like leveraging mentorship opportunities between facilities and integrating NCD focal persons within healthcare quality improvement teams to ensure consistent supervision. In Malawi, the Lighthouse Trust has documented its experience leveraging facility reviews in ART provision to improve service provision for hypertension clients in both clinical and non-clinical settings [43]. Such experience could be built upon, including supporting further local research to innovate and expand local capacity for NCD integration.

#### 4.2.2. Approaches to Optimize Skills Among Motivated yet Chronically Understaffed PHC Workforce

Despite shortages in established health personnel, all NCD clinics assessed met locally established personnel requirements (two staff per clinic day) on paper. Facility management and personnel also expressed motivation to provide NCD services. In practice however, we identified perceptions of NCDs as “extra work burden” among some health personnel in both country contexts. This reality threatens the prioritization and quality of NCD services delivered by an already overburdened health workforce. Like Zambia and Malawi, other SSA countries have reported chronic understaffing in lower level PHC settings to meet increasing demands for NCD services [44]. Alongside a lack of NCD-related CME, our study highlighted a discrepancy in training versus involvement of the few NCD-trained personnel, typically clinicians involved in administrative and other non-clinical tasks besides direct patient management. This task shifting or task sharing approach among human resources for optimized service delivery is not a new concept. It was extensively utilized during the height of the HIV/AIDS epidemic in Africa and has been sustained in contexts with acute personnel shortages [45,46,47]. The human resources density in our study contexts are significantly lower than both national and global benchmarks, therefore, existing task shifting approaches for NCDs should take into account the level of sophistication of tasks and corresponding skills required to accomplish them. In one facility in Zambia, this approach was already in use, whereby roles of community health workers involved in community health promotion services for HIV/AIDS were repurposed for supportive roles in health facilities during outpatient clinic days. The benefits of this approach has been documented in one study intervention in rural Malawi [42], which utilized integrated care clerks and expert patients for administrative and patient support, training and mentorship roles within poorly resourced integrated chronic care settings. As part of local efforts to attain universal healthcare coverage, we recommend scaling up such approaches in our study context and other similar settings. Alongside patient self-management strategies [48], this could help to offset the burden of personnel shortages and reduce barriers along the patient flow pathway by promoting uniformity, reducing stigma and harmonizing tasks among cadres rather than creating new ones. More importantly, it can help to promote a united vision to guide health workers and community gatekeepers for NCD-related preventive, promotive, curative and rehabilitative approaches that cascade along the continuum of service levels from primary care (including community) to tertiary and specialized care [49,50].

#### 4.2.3. Operationalization of NCD Surveillance and Reporting Mechanisms in PHC Settings

These results build on existing evidence that capacity building for NCD data management remains an urgent area of need. Separate studies have highlighted challenges in implementing integrated disease surveillance approaches that incorporate NCDs in low-income settings [51,52,53]. In both Malawi and Zambia, national documents reflect efforts to incorporate NCDs into national reporting via essential health package frameworks [14,15]. At operational level however, reporting systems for NCDs in both country settings lag behind in their level of sophistication when compared to HIV/AIDS, RMNCH conditions and common communicable diseases. In Zambia, NCDs were yet to be fully incorporated into the electronic medical records systems used in the facilities, due to end-user limitations in generating NCD-related reports from the current system in use (SmartCare). In Malawi, respondent accounts highlighted challenges in the uptake of facility-level reporting for NCDs into district or national decision-making. As at the assessment period, NCD data flow process in both contexts lacked clear standardized mechanisms in place. Installing electronic information systems in PHC facilities that includes NCD specifications could help to improve patient monitoring, quality of service and patient medical data and streamline easier uptake into multi-level health management information systems (HMIS) [54]. To mitigate this, local health authorities and health managers could incorporate NCD data management into continuous personnel training curricula could also help to promote data integrity and enhance its uptake for operational efficiency and informed decision-making.

#### 4.2.4. Moving from Episodic to Integrated Chronic Care Models

Our study established that the push for operationalizing integrated services for NCDs at PHC level in both countries is still in early stages (only 12–24 months operational). In Malawi, a study assessing the concept of chronic care clinics concluded that it is feasible to manage NCD patients in local primary care settings, but more is needed to improve clinic attendance and the control of hypertension and diabetes [55]. In Zambia, Aantjes et al. [54], note that a further reorientation of the Zambian health system is required to expand HIV/AIDS care as a basis to address NCDs. From our findings in Zambia, the PHC facility that showed highest preparedness for NCD service delivery also had the highest proportion of clients utilizing its ART services. All assessed PHC sites in this study form part of networks of recognized centres of excellence for HIV/AIDS care, both public-funded (Malawi) and faith-based (Zambia). As at assessment period, the nature of their services had expanded beyond exclusive HIV/AIDS services to include other services like RMNCH and NCDs for non-ART clients to meet increased demand. Multiple studies [42,56,57] have alluded to the co-benefits for NCD management within HIV/AIDS-led healthcare establishments, in terms of quality of care outcomes. Another growing realization among researchers is that, commonly cited and often “northern conceptualized” chronic care service models often have limited relevance in Saharan African countries as they operate on presumption of presence of optimum resources and well-developed health systems [54]. Our study has demonstrated the complexities of two health systems delivering NCD services under less than optimum conditions. This study also contributes to the necessary documentation through research, of existing challenges and milestones of integrated NCD service models within the realities of the local PHC contexts. Future interventions and research informed by these baseline results could be an opportunity for further operational guidance in the assessed sites. Subsequently, contextually relevant chronic care service models could be developed, that could influence decision-making in broader district or national efforts. As such, we agree that in addition to contextualizing models rather than a one-size-fits all approach, improving the quality of existing NCD services in a locally responsive manner will subsequently increase proportions of the population and especially young people in SSA, who begin to value them and therefore utilize the services.

### 4.3. Limitations and Strengths

This study aimed to assess the current state of integration of NCD services in operational settings within selected resource-limited primary care settings with the aim to identify areas of further support. We acknowledge the limitation in generalizability of the results of our study sites as they are non-randomly selected. However, it seems unlikely that on average, other similar facilities would perform significantly better since despite all aforementioned shortcomings, the facilities represented in this study get some external support and are attached to designated centers of excellence in both countries. The assessed Malawi sample sites included in this study for example, also represent 67% service coverage (2019) in terms of the number of government-supported primary health facilities delivering NCD-related services in Lilongwe district, hence real world relevance of the study results. Additionally, since 2010, only 28% of SSA countries have conducted recent national or sub-national surveys of health facilities to assess service availability and readiness for NCDs. As opposed to generalized surveys, few of such studies also explore in-depth assessments of processes at individual facility level [21]. We adopted and reorganized our study questionnaire sections from validated tools and methodologies of the WHO and respective MoH, and after consultations with local experts, we piloted them for local relevance. We acknowledge however, that this process could have also benefited from an extra validation step such as the utilization of a content validity index to rank process measures.

Separate studies [58,59], in both Malawi and Zambia have highlighted some existing gaps in translating national NCD policies to action within the respective national health systems, with a rallying call to build more locally relevant evidence to inform decision-making alongside innovative cost-effective solutions. The insights offered into the operational challenges faced when translating policy directives in a public healthcare setting. As NCD care is often complex and the services in our target sites are still in early stages, establishing feasible operational elements that are responsive to both the needs of the PHC settings and the communities they serve is paramount. With glaring human resources for health challenges in both countries [60,61], understanding the context within which the existing health workforce operates could also help to leverage their inputs into better allocation of the limited resources and subsequently promote better health outcomes.

## 5. Conclusions

In providing a comprehensive review of the current state of NCD service integration in health networks in Zambia and Malawi, this study confirms the challenges of modifying complex health systems. The existing efforts in Malawi and Zambia to integrate NCD services at policy level and primary care level, signify a demand for the services and their increasing recognition as an important public health issue. The current set-up of integration of NCD services within the assessed PHC contexts are in early stages and coupled with capacity gaps and challenges, requiring further operational guidance and support. As a result, based on benchmark requirements, the assessed PHC sites were generally not prepared to offer NCD services. However, existing motivation among healthcare staff to provide NCD services in spite of severe resource limitations and training gaps presents an opportunity for policy makers, subject experts and program managers in the respective country contexts to respond accordingly.

While these study findings are underpinned by baseline assessments, there are early lessons that future research and policy in the two country contexts and other similar settings could build upon as follows:

1. *Zambia:* Health managers and policy makers could build on these findings to further deliberate the inclusion of quality NCD indicators in existing routine electronic reporting and decision making at all levels. Future research could explore patient experiences, both ART and non-ART in accessing integrated services at similar points of care, and effects on quality-of-care outcomes.
2. *Malawi:* Strengthen the coordination mechanism within the health system to improve feedback and uptake of multi-level reporting for NCDs, as well as interfacility linkages. Integrate NCDs in sub-national health plans and quality improvement teams. Integrate NCD training for non-clinical personnel.
3. *Both:* The multi-country collaborative spaces established by the existing healthcare networks and their recognition by the respective country governments could be leveraged to further engage multi and intersectoral networks within and outside the health sector and build momentum for a more coalesced, evidence-driven NCD service delivery agenda. Within countries, the respective organizations and study sites could use experiences gathered to mentor other PHC networks and contribute to the scale up of district and national efforts for NCD prevention and control. Future research could delve deeper into thematic issues within individual service delivery components, e.g., NCD-related human resources, effectiveness of health promotion and preventive services for NCDs, in-depth analyses of drug supply challenges and further implementation research to assess effectiveness of NCD-specific interventions.

## Figures and Tables

**Table 1:** Characteristics of Assessed PHC network sites and respondents.

Facility Characteristics	Lilongwe, Malawi *n* = 4	Lusaka, Zambia *n* = 4	* ZaMaC sites N = 8
Classification by type			
Urban	2 (50%)	3 (75%)	5 (62.5%)
Rural	2 (50%)	1 (25%)	3 (37.5%)
Catchment Population (as at 1 January 2019)			
Range:	98,000–310,200	3000–197,000	3000–310,200
IQR:	108,000–285,900	25,750–197,000	95,000–209,000
Median:	177,900	145,500	167,500
By distance to nearest referral facility (km)			
IQR:	4.8–38.3	2.3–15.1	3.5–29.8
Median:	21.5	7.5	10
Priority NCD services			
CVD (mainly hypertension)	4 (100%)	4 (100%)	8 (100%)
Diabetes mellitus	3 (75%)	3 (75%)	6 (75%)
COPD/asthma	4 (100%)	4 (100%)	8 (100%)
Cervical cancer screening	1 (25%)	1 (25%)	2 (25%)
Point of integration along patient flow			
General outpatient	3 (75%)	3 (75%)	6 (75%)
As a separate integrated clinic	4 (100%)	1 (25%)	5 (62.5)
Within ART services	4 (100%)	4 (100%)	8 (100%)
Other	2 (50%)	2 (50%)	8 (100%)
Respondent Characteristics	*n* = 16	*n* = 4	*n* = 20 *
Sex			
Female	9 (56.3%)	2 (50%)	11(55%)
Male	7 (43.8%)	2 (50%)	9 (45%)
Age			
Below 29	3 (18.8%)	0 (0.00%)	3 (15%)
30–39	11 (68.8%)	3 (75%)	14 (70%)
40–59	2 (12.4%)	1 (25%)	3 (15%)
By education			
University (4 years or more)	7 (43.8%)	3 (75%)	10 (50%)
College (3 years or less)	9 (56.3%)	1 (25%)	10 (50%)

* ZaMaC = Zambia–Malawi Collaboration on NCDs Project PHC sites combined.

**Table 2:** Summary comparison of facility capacity by country network versus service domains.

Category	** Expected Score %	** Required Score %	Malawi (Lighthouse PHC Sites) *n* = 4 Average (Range: Min-Max)	Zambia (CHRESO PHC Sites) *n* = 4 Average (Range: Min-Max)	* ZaMaC Sites N = 8 (Mean + Sample SD)
General measures and infrastructure score as% out of 6	100	66.7	81.3 (75.0–91.7)	87.5 (58.3–100.0)	84.38 ± 14.39
Essential drugs availability score as% out 18	100	77.8	68.1 (66.7–72.2)	58.3 (44.4–77.8)	63.19 ± 11.08
Basic medical equipment as% out of 18	100	66.7	63.9 (55.6–88.9)	56.9 (44.4–66.7)	60.41 ± 13.09
Diagnostic capacity as% out of 26	100	65.3	64.4 (55.9–75.7)	66.3 (57.7–73.1)	65.38 ± 8.54
Process and quality measures as% out of 24	100	66.7	40.1 (27.0–47.0)	55.2 (43.8–72.9)	47.66 ± 13.48
Total NCD Service capacity score%	100	67.9	59.0 (52.6–69.2)	63.1 (50.6–73.7)	61.06 ± 8.24
HF Preparedness benchmark achieved (Yes/No)	Yes	Yes	No	No	No

* ZaMaC = Zambia–Malawi Collaboration on NCDs Project PHC sites combined; ** expected and required domain scores further detailed in Table A1.

**Table 3:** Respondents (health personnel) perspectives of NCD services.

Themes	Verbatim Quotes from Qualitative Interviews	Respondent
NCD-related medicine supply and value chain	“The essential NCD drugs purchased are decided by the district health office and often times facility-specific needs are ignored.” “The paper-based stock management for drugs and medical supply is cumbersome”	-T5R1 Pharmacy-in-Charge #1 (Lilongwe)
	“Our procurement system is too slow. It can take more than a week in case of emergencies. Anti-asthmatic drugs run out fast during the cold season but are not stocked in time. We also have a “push system” where some drugs from other facilities may be brought shortly before expiring and don’t move fast enough hence, they expire on shelves.”	-T5R2 District Health Office (Lilongwe)
	“Our supply of NCD drugs from the government is erratic and barely meets the demand. We experience frequent stockouts of antihypertensives and oral hypoglycemics. We also have no insulin.”	-T5R3 Health Worker #1 (Lusaka)
Human resource availability vs. service utilization	“It is now 10.30 am already and my colleague who is supposed to be at the General outpatient clinic has not yet shown up. I am therefore the only clinician on duty today and I have to step in as the numbers in general OPD are obviously very many. That means NCD clients at the clinic will most likely not be attended to today or they will have to wait very long.”	-T5R4 NCD Focal Person #1 (Lilongwe)
Patient flow management	“When a patient arrives, they give client cards to community health workers who pull out their files and direct them to the vital waiting area. During first consultation, the patient will be screened, diagnosed, given counselling and treatment and referred if need be. Unfortunately, non-ART NCD clients are not captured in the electronic records system so it is a bit more difficult to monitor and follow-up compared to ART clients.”	-T5R5 NCD Focal Person #2 (Lusaka)
Human resource training versus involvement	“NCDs for us here even though we know it’s a problem, it is currently a one-man clinic by one medical assistant. Even within the facility, other staff are not involved. When NCD drugs go out of stock, if the pharmacy technician is not pushed, they will not put it as a priority. The big issue here is knowledge and we need to include all staff, including support staff in training.”	-T5R6 Facility-in-Charge #1 (Lilongwe)
Health worker attitudes and perspectives on operationalizing NCD integration	“Integration should be responsive to all the needs of the patient at the point of care without causing too much burden to the health worker. With more support and training, the services will keep improving.”	-T5R7 Facility-in-Charge #2 (Lusaka)
	“For me integration means a one stop shop where when a client comes to the facility for any service at any department or clinic, they are still able to receive any specific service needed at that point, including NCDs. I do not see why all clinics would not be able to screen for hypertension and diabetes for example.”	-T5R8 Facility-in-Charge #3 (Lilongwe urban)
	“Currently as a facility we are supposed to be providing the NCD services for hypertension, diabetes and Asthma but we have no guideline. We have to develop our own, but as clinicians, we are motivated to provide the services, we just lack resources here. ”	-T5R9 Facility-in-Charge #4 (Lilongwe rural)

## Data Availability

The authors confirm that most of the data supporting the findings of this study are available within the article and its appendices. Raw numerical data were generated at the respective health facilities included in the study. However, as this is an ongoing study, some of the data is restricted due to information that could compromise the privacy of research participants and ongoing investigations.

## Appendix A

**Table A1:** Facility Capacity and Preparedness Assessment Matrix.

General Measures and Infrastructure (G) **	Human Resource Availability (H) **	Drug, Laboratory and Equipment Availability and Measures (ELQ)			Process/Quality Measures (RFPT)			
		Essential Drugs Supply (by Group Type) (E) **	Laboratory (L) **	Basic Medical Equipment (Q) **	Record Keeping/HMIS (R) **	Patient Flow Optimization (F) **	Essential Protocols & IEC Materials (P) **	CME & Training (T) **
- Facility/clinic operational hours (+ communication lines) - Availability of electricity - Infection prevention and control (IPC) - Consultation rooms for NCD clients - Furniture (chair, desk and examination bed)-Temperature controlled storage	- NCD-designated medical officer or physician * - NCD-designated clinical officer or medical assistant - NCD-designated nurse - NCD-designated non-clinical support staff	- Diuretics - ACE-inhibitors - Beta blockers - Calcium channel blockers - Blood thinners - Glyceryl trinitrate - Oral hypoglycemics - Insulins - Bronchodilators - Antibiotics - Steroids/corticosteroids - Anti-emetics - Pain management - Constipation - Glucose injection/solution - Sodium chloride infusion - Vitamin B (Pyridoxine) - Statins	- Can lab perform full blood count? - Blood glucose test strips - -Urine dipsticks (ketones, protein and glucose) - HIV testing kits - Urine microalbuminuria test strips - Working microscope - Serum creatinine assay * - Blood cholesterol test kits *	- Functioning BP apparatus - Adult weighing machine - Stethoscope - Measurement tape - Height board - Thermometer - Peak flow meter - Spacers for inhalers - Glucometer - HbA1c machine - Visual Acuity Board - Ambu-bag - Patella hammer/tuning fork - Syringes and needles - Nebulizer * - Pulse oximeter * Ophthalmoscopy - Administration of oxygen (via mask or tube) *	- Operational electronic or manual registry - Available and updated NCD patient clinical records - Available and updated drug stock cards - Available and updated laboratory stock cards - Equipment maintenance records - Facility-wide review of mortality records - Frequency of audits of medical records or registers for SOP compliance	- Visual flow chart with referral criteria - One stop registration, vital check and triaging - Adequacy and ventilation of designated waiting area(s) - Visual and auditory privacy in consultation - Day bed/ward - Emergency transport on standby 24 hours - IN/OUT referral documentation available and in use - Appointment and client follow-up/ feedback system - Active NCD patient support group(s) or program at facility or community	- Copies of national treatment guidelines - Evidence-based clinical standard operating procedures (SOPs) - Risk stratification charts available and/or in display - Information, education and communication (IEC) materials for NCD in display - Health education roster	- NCD-trained clinicians last 24 months - NCD-trained nurses last 24 months - NCD-trained clinic support staff last 24 months - NCD-designated mentorship
6 items,	4 items,	Items, 18	Items, 8	Items, 18	Items, 7	Items, 9	Items, 4	Items, 4
Expected score = 6	Expected score = 4	Expected score = 18	Expected score = 8	Expected score = 18	Expected score = 7	Expected score = 9	Expected score = 4	Expected score = 4
Required value, V_G_ = 4 (66.7%)	Required value, V_H_ = 2 (50%)	Required value, V_E_ = 14 (77.8%)	Required value, V_L_ = 5 (62.5%)	Required value, V_Q_ = 12 (66.7%)	Required value, V_R_ = 5 (71.4%)	Required value, V_F_ = 5 (55.6%)	Required value, V_P_ = 4 (100%)	Required value, V_T_ = 2 (50%)

* Check for availability of equipment if facility is physician or medical officer-led. ** Service domains = G, H, E, L, Q, R, F, P and T. Total items/measures, G + H + E + L + Q + R + F + P + T= 78 (100%). Calculated preparedness benchmark value = mean sum of required values (V_G_ + V_H_ + V_E_ + V_L_ + V_Q_ + V_R_ + V_F_ + V_P_ + V_T_) as % of total items, = 53 (69.7%). Service domain score = mean availability of items per domain as %. Capacity score = sum of average weighted scores for service-specific preparedness as %.
